# Pulsed oral sirolimus in advanced autosomal-dominant polycystic kidney disease (Vienna RAP Study): study protocol for a randomized controlled trial

**DOI:** 10.1186/s13063-015-0692-3

**Published:** 2015-04-23

**Authors:** Markus Riegersperger, Harald Herkner, Gere Sunder-Plassmann

**Affiliations:** Department of Medicine III, Division of Nephrology and Dialysis, Medical University of Vienna, Währinger Gürtel 18-20, A-1090 Vienna, Austria; Department of Emergency Medicine, Medical University of Vienna, Währinger Gürtel 18-20, A-1090 Vienna, Austria

**Keywords:** Autosomal-dominant polycystic kidney disease, ADPKD, *PKD*, Sirolimus, Mammalian target of rapamycin, mTOR, mTOR-inhibition, Pulsed, Rapamycin

## Abstract

**Background:**

Autosomal-dominant polycystic kidney disease (ADPKD) is a hereditary illness that causes renal tubular epithelial cells to form cysts that proliferate and destroy renal tissue. This usually leads to a decline in renal function, and often to terminal kidney failure, with need for renal replacement therapy. There is currently no causative therapy. The mammalian target of rapamycin (mTOR) inhibitor sirolimus (SIR) is an immunosuppressant with strong antiproliferative effects, and is potentially able to stop or reduce cyst growth and preserve renal function in ADPKD. Continuous mTOR exposure results in a loss of its antiproliferative effects on renal tubular cells. With a half-life of roughly 60 hours, pulsed (weekly) administration of SIR may be an effective way to reduce cyst growth and preserve excretory renal function in ADPKD.

**Methods/Design:**

The Vienna RAP Study is a randomized, double-blind, placebo-controlled trial, funded by the Anniversary Fund of the Oesterreichische Nationalbank. We will investigate the effects of a weekly dose of 3 mg SIR on kidney function in 34 patients with advanced ADPKD, compared to a placebo equivalent in 34 patients with advanced ADPKD, over 24 months. The primary endpoint is creatinine level (less or equal than 1.5-fold increase in serum creatinine without initiation of dialysis over two years) and dialysis, renal transplantation, or death. The secondary endpoints are safety, change in proteinuria (as indicated by albumin/creatinine- and protein/creatinine ratio, respectively), and creatinine clearance.

**Discussions:**

The Vienna RAP Study is, to the best of our knowledge, the first study to investigate the effects of a pulsed (weekly) dose of SIR on renal function in ADPKD.

**Trial registration:**

This trial was registered with EudraCT (identifier: 2012-000550-60 (EU)) on 27 November 2013 and with ClinicalTrials.gov (identifier: NCT02055079 (USA)) on 3 February 2014.

## Background

Autosomal-dominant polycystic kidney disease (ADPKD) is a genetic disorder characterized by uncontrolled proliferation of innumerable macro- and microscopic epithelial-lined cysts that stem from renal tubular cells. They compress and destroy renal tissue which leads to a gradual decline in renal function. Other than supportive care, there is no treatment, and most often renal replacement therapy by either dialysis or kidney transplantation is necessary. ADPKD is detected by kidney ultrasound [1-3], or computed tomography or magnetic resonance imaging. Depending on whether the mutation is located on the *PKD1* or *PKD2* gene, ADPKD 1 or 2 respectively, develops. In the *PKD1* mutation, patients usually present in the third to fourth decade, and renal replacement therapy usually becomes necessary in the following decade. In the *PKD2* mutation, patients present around the fifth decade, and often experience a milder course [4]. Effects of the immunosuppressant sirolimus (SIR) on cyst growth in ADPKD have been developed in a rodent model with Han:SPRD rats (Cy/+). SIR applied intraperitoneally leads to a reduction of overall kidney size, a decrease in cyst density, and tubular cell proliferation [5]. SIR applied orally reduced worsening of kidney function, cyst proliferation, cyst volume, and cyst density [6]. mTOR inhibition (mTOR-I) by either SIR or everolimus (EVER) has been investigated in preclinical studies and clinical trials but only subtle, if any, clinically relevant effects on cyst growth and the preservation of renal function were found [7,8]. Tubular cells, the target of mTOR-I in ADPKD, develop a resistance towards SIR *in vitro* as well as *in vivo* [9]. In a rodent model of the influence of SIR on the proliferation of renal tubular cells in acute renal failure, continuous exposure with SIR had a strong anti-proliferative effect throughout the first three days, which drastically decreased throughout the fourth to sixth day. The half maximal inhibitory concentration (IC50) of SIR increased from approximately 10 ng/mL to approximately 100 ng/mL within a week of exposure. Until now, no study has accounted for the loss of the strong anti-proliferative effects of SIR after the fourth day of continuous exposure.

### Aim of the trial

The aim of this trial is to disprove the null hypothesis that pulsed administration of the mTOR-I SIR in a fixed weekly oral dose of 3 mg compared to placebo does not preserve excretory renal function in patients with ADPKD and an estimated glomerular filtration rate (eGFR) below 60 mL/min per 1.73 m^2^.

## Methods/Design

### Study design, approval, and registration

The Vienna RAP Study is a randomized, placebo-controlled, double-blind, single-center trial. Treatment for both active and placebo groups will be for 24-months duration. The study is funded with €70,000 provided by the Anniversary Fund of the Oesterreichische Nationalbank (project grant number 15170). The study was approved by the Ethics Commission of the Medical University of Vienna (identifying number 1060/2012). The study was registered at the Competent Austrian Authorities, Bundesamt für Sicherheit im Gesundheitswesen (identifying number LCM-718208-0001), at the European Medicines Agency EudraCT (identifying number 2012-000550-60), and at the United States Institute of Health ClinicalTrials.gov (identifying number NCT02055079).

### Participants and site recruitment

Patients with ADPKD and an eGFR (4-variable modification of diet in renal disease (MDRD) equation) below 60 mL/min per 1.73 m^2^ will be included at the outpatient clinic of the Division of Nephrology and Dialysis, Department of Medicine III, Medical University of Vienna by the principal investigator and his representatives. The diagnosis will be confirmed by imaging as stated above, there will be no discrimination of *PKD1* or *PKD2* within the study population. Subjects must sign the informed consent form to be included in the study. See Table 1 for inclusion and exclusion criteria. Familial medical history and all relevant demographic data will be recorded.

**Table 1:** **Subject inclusion and exclusion criteria of the Vienna RAP Study**

**Inclusion criteria**	
	• ADPKD, as confirmed by history, ultrasound, computed tomography or magnetic resonance imaging
	• 18 years of age, or older
	• Baseline eGFR (4-variable MDRD equation) below 60 mL/min per 1.73 m^2^
	• Negative serum pregnancy test prior to administration of sirolimus and agreement to use contraception throughout the study and for three months after
	• Written informed consent
**Exclusion criteria**	
	• Need for renal replacement therapy
	• Pregnancy or lactation
	• Plans to become pregnant in the near future
	• Refusal to use sufficient contraception
	• Proteinuria as defined as protein:creatinine ratio >1,000 or >1 g/d, respectively
	• History of life-threatening complications of ADPKD
	• Evidence of active systemic or localized major infection
	• Evidence of infiltrate or consolidation on chest X-ray
	• Use of any investigational drug or treatment up to four weeks prior to enrolment and during the study
	• Known allergy or hypersensitivity to sirolimus and its derivatives
	• Medication that will interfere with the CYP3A4/CYP3A5 system
	• Total white blood cell count below or equal to 3,000/mm^3^
	• Platelet count below or equal to 100,000/mm^3^
	• Fasting triglycerides above or equal to 400 mg/dL
	• Fasting total cholesterol above or equal to 300 mg/dL
	• Concomitant glomerular diseases
	• Psychiatric disorders and any condition that might prevent full comprehension of the purposes and risks of the study
	• History of malignancy, with the exception of adequately treated basal cell and squamous cell carcinoma of the skin
	• HIV positivity

ADPKD, autosomal-dominant polycystic kidney disease; CYP, cytochrome p-450; eGFR, estimated glomerular filtration rate; HIV, human immunodeficiency virus; MDRD, modification of diet in renal disease.

### Enrollment and randomization

Randomization will be performed independent of patient recruitment or inclusion. To yield balanced yet unpredictable groups, we will use a block-randomization scheme in varying blocks of four to six. This randomization list will be provided to the hospital pharmacy for the preparation of the blinded study drugs and otherwise concealed until analysis of the study. Patient ID will be linked to container numbers during the course of the study to warrant allocation concealment. For individual code breaking, we will prepare a set of sequentially numbered sealed opaque envelopes that contain information about treatment allocation. At the end of the study all envelopes will be checked to assess the absence of code breaking. All code breaks will be documented in detail by the study investigators. The randomization code will be broken in the case of any serious adverse event (SAE) if knowledge of the study drug is relevant to the treatment of the SAE. A consecutive number will be assigned to any subject partaking in the study as unique patient identifier code at the time of inclusion.

### Interventions

The treatment in arm one will be a weekly oral dose of 3 mg SIR (Rapamune®, Pfizer Ireland Pharmaceuticals, Little Connell, Newbridge, Co. Kildare, Ireland), or placebo (Manufactured at the hospital pharmacy; maltodextrin powder in bovine empty hard gelatine capsules, Capsugel®, Bornem, Belgium) equivalent in arm two (both blinded). SIR oral loading doses of 6 mg are well tolerated in recipients of renal transplants. Pulsed intravenous admission of the SIR analog temsirolimus at doses of 25 mg per week was shown safe and effective in patients with renal cell carcinoma and hematological malignancies [10]. Since SIR effects mTOR inhibition at very low doses, we will administer a pulsed oral dose of 3 mg per week without the necessity to adjust trough levels. To minimize the risk of accidental intoxication, SIR trough levels will be measured at every visit. These measurements will only be accessible for nominated nephrologists not otherwise involved in the study, to protect blinding of the treating physicians and the patients. The nominated nephrologists will notify the treating physicians in case of trough levels exceeding 20 ng/mL.

### Study regimens

**Figure 1:**
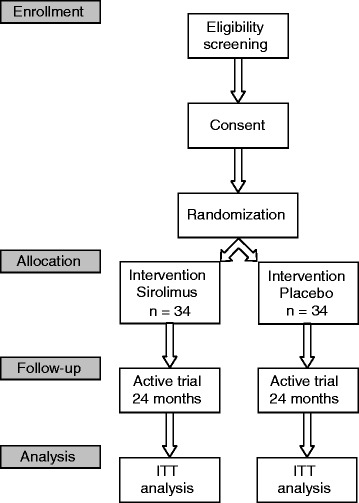
Patient flow of The Vienna RAP Study. ITT, intention to treat.

See Figure 1 and Table 2 for the patient flow and trial sequence.

**Table 2:** **Trial sequence of The Vienna RAP Study**

**Timeline**	**E**	**W1**	**W2**	**M1**	**M6**	**M12**	**M18**	**M24**
**Visit**	**V1**	**V2**	**V3**	**V4**	**V5**	**V6**	**V7**	**V7**
**General**								
Demography	•							
Clinical check	•				•	•	•	•
Chemistry								
Sodium	•			•	•	•	•	•
Potassium	•			•	•	•	•	•
Calcium	•			•	•	•	•	•
Phosphate	•			•	•	•	•	•
Creatinine	•	•	•	•	•	•	•	•
Urea	•			•	•	•	•	•
ASAT	•			•	•	•	•	•
ALAT	•			•	•	•	•	•
*γ*-GT	•			•	•	•	•	•
Lipids	•			•	•	•	•	•
Hba1c	•			•	•	•	•	•
Glucose	•			•	•	•	•	•
Pregnancy test	•			•	•	•	•	•
Sirolimus trough level^a^		•	•	•	•	•	•	•
**Hematology**								
Hemoglobin	•			•	•	•	•	•
Leucocytes	•	•	•	•	•	•	•	•
Platelets	•	•	•	•	•	•	•	•
**Urine**								
24 hour proteinuria	•				•	•	•	•
Creatinine clearance	•				•	•	•	•
Protein/creatinine ratio	•	•	•	•	•	•	•	•
Albumin/creatinine ratio	•	•	•	•	•	•	•	•
**Immunology**								
HBV	•							
HCV	•							
HIV	•							
**Vitamin D status**	•				•	•	•	•
**PTH**	•				•	•	•	•
**Nutritionist counseling**	•							

ALAT, alanine aminotransferase; ASAT, aspartate aminotransferase; E, enrolment; γ-GT, gamma glutamyl transferase; HbA1c, glycated hemoglobin; HBV, hepatitis B virus; HCV, hepatitis C virus; HIV, human immunodeficiency virus; M, month; PTH, parathyroid hormone; V, visit W week; . ^a^To minimize the risk of accidental intoxication, sirolimus trough levels will be measured at every visit. These measurements will only be accessible for nominated nephrologists not otherwise involved in the study to protect blinding of the treating physicians and the patients. The nominated nephrologists will notify the treating physicians in case of trough levels exceeding 20 ng/mL.

### Dispensing, blinding, and monitoring of adherence

SIR will be provided by the manufacturer free of charge. The placebo will be produced and all study medication will be blinded by the pharmacy of the Vienna General Hospital. The blinded medication will be dispensed in sealed opaque containers, labeled with continuous study IDs, by the principal investigator and his representatives. Subject adherence will be monitored by interview at study check-up visits.

### Concomitant medication during the trial

SIR is extensively metabolized by the hepatic and intestinal isozyme cytochrome p-450 (CYP3A4). It is also a substrate for the multidrug efflux pump P-glycoprotein (P-gp), located in the small intestine. Curative or supportive medication may be co-administered during the trial without hesitation, with the exception of CYP3A4 inhibitors and inducers (Table 3), where alert monitoring for side effects is necessary in the former.

**Table 3:** **Cytochrome p-450 inhibitors and inducers**

**CYP3A4 inhibitors:**	**CYP3A4 inducers**
• Bromocriptine	• Carbamazepine
• Cimetidine	• Rifabutin
• Clarithromycin	• Rifampicin
• Danazol	• Phenobarbital
• Fluconazole	• Phenytoin
• Itraconazole	• St John’s Wort (*Hypericum perforatum*)
• Protease inhibitors	
• Telithromycin	

To avoid potential intoxication or reduced efficacy by accidental co-administration of these substances, every participant will be handed a list of the medication above for emergency use.

### Standardized care

To ensure standardized care of disease progression modifying factors, blood pressure will be targeted at <130/80 mmHg, according to the Task Force for the Management of Arterial Hypertension of the European Society of Hypertension (ESH) and the European Society of Cardiology (ESC) [11], in both treatment arms. Since the optimal parathyroid hormone (PTH) levels in patients with chronic kidney disease (CKD) stages three to five not on dialysis are not known, the Kidney Disease Improving Global Outcomes (KDIGO) Clinical Practice Guideline for the Diagnosis, Evaluation, Prevention, and Treatment of Chronic Kidney Disease - Mineral and Bone Disorder (CKD-MBD) suggests that patients with levels of intact PTH above the upper limit of normal of the assay are first evaluated for hyperphosphatemia, hypocalcemia, and vitamin D deficiency. Therapy should include the reduction of dietary phosphate intake and the administration of phosphate binders, calcium supplements, and/or native vitamin D. In patients with CKD stages three to five not on dialysis, in whom serum PTH is progressively rising and remains persistently above the upper limit of normal for the assay despite correction of modifiable factors, treatment with calcitriol or vitamin D analogues is suggested [12]. In participants of this study target PTH levels are defined as 2- to 3-fold of the normal range (15 to 65 pg/mL). The pH level as identified by venous blood sampling is defined as 7.35 to 7.43, and bicarbonate target levels are defined as ≥20 mmol/L. Also, every participant will receive dietary advice by a nutritionist at the trial site at enrollment. Common side effects will be monitored and documented where appropriate, see Table 4.

**Table 4:** **Commonly reported adverse reactions of sirolimus therapy (occurring in more than 10% of patients)**

•	Abdominal pain
•	Acne
•	Anemia
•	Aphthae
•	Arthralgia
•	Constipation
•	Diarrhea
•	Headache
•	Hypercholesterolemia
•	Hyperglycemia
•	Hypertension
•	Hypertriglyceridemia
•	Hypokalemia
•	Hypophosphatemia
•	Impaired wound healing
•	Increased serum creatinine
•	Increased serum lactate dehydrogenase
•	Nausea
•	Pain
•	Peripheral edema
•	Pneumonitis
•	Pyrexia
•	Stomatitis
•	Thrombocytopenia
•	Urinary tract infection

### Trial termination and post-trial care

The trial will be terminated per protocol when the last patient finishes the trial period of 24 months. Any patient who wishes to discontinue the trial prematurely may do so without providing a reason. All individuals who discontinue the trial will re-enter the outpatient program at the outpatient clinic of the Division of Nephrology and Dialysis, Department of Medicine III, Medical University of Vienna. Given the low dose treatment and the small sample size, we do not plan a preterm stopping of the trial.

### Endpoints

The primary endpoint is a 50% reduction in the doubling of serum creatinine, or initiation of dialysis, renal transplantation, or death over a period of 2 years. A 1.5-fold or lower increase in serum creatinine without initiation of dialysis, renal transplantation, or death over 2 years is considered a beneficial outcome; increases in serum creatinine greater than 1.5 over 2 years or initiation of dialysis, renal transplantation, or death are considered a non-beneficial outcome. Secondary endpoints are safety, change in proteinuria (as indicated by albumin/creatinine- and protein/creatinine ratio, respectively), and creatinine clearance.

### Method of creatinine determination and estimation of glomerular filtration rate

The serum creatinine values are analyzed by the enzymatic CREJ2 cobas® Jaffé Gen. 2 essay (Roche Diagnostics GmbH, Mannheim, Germany) traceable to isotope dilution mass spectroscopy (IDMS). The eGFR will be calculated by the 4-variable MDRD equation [13]. Additional estimates of the GFR will be made by the Chronic Kidney Disease Epidemiology Collaboration (CKD-EPI) formula [14]. The results of these two estimation methods will be compared to analyze the concordance of performance.

### Measures to reduce influences of creatinine variability on endpoints

To reduce the impact of fluctuations and variability of serum creatinine on primary endpoint results, we will perform repeated measurements of serum creatinine (and estimations of GFR) at the end of the study at month 24, and define the median value of four repeated measurements (once every week over a period of four weeks) as the creatinine value at the end of the study. To ensure standardized conditions, we will give instructions to patients to maintain well hydrated, and to avoid excessive consumption of meat and rigorous physical activity prior to blood sampling.

### Source data, data collection, and documentation

The participants’ medical records are to be considered the source data. The data for the statistical analysis will be entered in electronic case report forms (eCRF), as provided by the Center for Medical Statistics, Informatics and Intelligent Systems (CeMSIIS) by the server-based platform Clincase Software Structure for Clinical Trials (Quadratek Data Solutions Ltd., London, UK). Data will be entered only by the unique identifier code; no patient names will be mentioned in the eCRF. The study nurse or study coordinator will be authorized to enter data. The principal investigator will have administrator rights to enter data and monitor data entry. Additional personnel may be authorized for data entry by the principal investigator, provided that the additional staff are registered at the competent authorities with an amendment.

### Subject withdrawal criteria and procedures

Any participant who becomes pregnant during the study period and every female subject of child-bearing potential who refuses to use sufficient contraceptives during the trial will have to discontinue. Any participant who develops *de novo* proteinuria, as defined as an elevation of protein/albumin:creatinine ratio >200 and >30, respectively, will have to discontinue the trial in case the protein:creatinine ratio exceeds the cut-off levels of >1,000 or >1 g/d, respectively. Any participant who refuses to follow the trial sequence will have to discontinue. Any patient who discontinued the study will re-enter follow-up at the outpatient clinic for routine check-up procedures. Withdrawn subjects will have to return the remaining study medication.

### Statistical methods

Categorical data will be presented as absolute and relative frequencies, and interval data will be presented as mean and standard deviation or median and interquartile range, as appropriate. Baseline data will be tabulated and compared qualitatively and quantitatively. The primary analysis includes an intention-to-treat comparison of the primary and secondary endpoints. The primary endpoint is dichotomous and will be treated accordingly. We assess the effect of the intervention by calculating a relative risk with a 95% confidence interval. To test the null hypothesis of no difference between the groups we will use the Fisher’s exact test. For the secondary endpoint change in proteinuria, as indicated by albumin/creatinine and protein/creatinine ratio, we will compare the individual regression slopes from baseline to study end in these variables between intervention and placebo group; we will calculate differences and 95% confidence intervals. For hypothesis testing, we will use an unpaired sample t test if a normal distribution can be sensibly assumed, otherwise the Mann-Whitney U test will be used. Adverse events (AE) will be dichotomized and analyzed like the primary endpoint. Secondary analysis will be performed if baseline differences should be noted. In this case we will use multivariable regression methods to adjust for such baseline differences. For data management, range and consistency checks, and analyses we will use Excel (Microsoft, Redmond, WA, USA) and Stata 11 (Stata Corp. College Station, TX, USA). No *a priori* subgroup analyses are planned. An interim analysis is not scheduled. To test for a possible dependency of GFR on the effect of SIR in these patients we will perform an explorative interaction analysis.

### Number of subjects and level of significance

The calculation of the sample size is based on the results of the most comprehensive follow-up of ADPKD patients with advanced kidney disease. With a mean baseline creatinine of 3 mg/dL, or a mean eGFR of 23 mL/min per 1.73 m^2^, respectively, 40.5% of 142 patients showed a doubling of serum creatinine or dialysis after a mean follow-up of 2.3 years [15]. However, with less than 1.5 fold increase instead of 2 fold increase in creatinine we used a stricter criterion for a favorable outcome, and therefore set our expected outcome probability in the control group at 50%. Formally 31 patients in each group are necessary to demonstrate a clinically meaningful reduction of 50 to 90% of the primary endpoint at an α = 0.05 with a power of 80%. To allow for loss to follow-up, we increase this formal sample size by 10%. Therefore we plan to include 34 patients per group or 68 patients overall into our study. Generally a probability (*P*) value of less than 0.05 is considered statistically significant.

### Procedure for accounting for missing, unused, and spurious data

Every effort will be made to avoid missing data. The primary endpoint was designed in a way that missing data is very unlikely. If data are missing, we will assess whether data are missing at random or not by looking for systematic differences between patients with complete datasets and those with missing values. If longitudinal data are missing at random, we will use a mixed-model approach. If missing not at random cannot be excluded, we will perform further sensitivity analyses following the principles outlined in a recent guidance paper [16]. Spurious data will be checked for transmission errors with the source data and investigated by the clinical epidemiologist (HH). To prevent event-triggered missing data, we will introduce GFR substitutes for the three failure events initiation of dialysis, renal transplantation, or death, with an adjusted expected eGFR of 5 mL/min per 1.73 m^2^ [17].

### Subjects to be included in the analyses

- Every individual who finishes the study will enter the per protocol analysis.
- Every individual with a single exposure to the drug will enter the intention-to-treat analysis.
- Every individual who declines to partake in the study after randomization and before the first exposure to the drug will be excluded of any analysis.

### Monitoring of cyst growth

Radiographic monitoring of cyst growth, especially magnetic resonance imaging cyst volumetry [18], has been proposed as a marker of disease progression [19]. Lately this concept has been challenged, since at least 50% of the cysts are undetectable by magnetic resonance imaging due to their size being below the radiographic and/or macroscopic threshold [20,21]. Therefore, we refrain from any form of radiographic kidney and cyst volumetry and define serum creatinine levels as the marker of therapeutic effects, since they directly represent the excretory kidney function. Retardation or termination of cyst growth inevitably results in a reduction or the halt of the decline in eGFR.

### Assessment of safety

Safety of SIR and other mTOR inhibitors in this population has been well established in numerous clinical trials. The principal investigator and the sub-investigator have conducted a pilot safety trial, Rapamycin in Advanced Polycystic Kidney Disease - The Vienna RAP Safety Pilot Study (EudraCT 2008-007980-18), approved by the Ethics Committee at the Medical University of Vienna (identifier 940/2009). In this trial 8 patients older than 18 years of age attending the outpatient department of the Division of Nephrology and Dialysis, Department of Medicine III at the Medical University of Vienna with advanced ADPKD and an eGFR of 20 to 40 mL/min per 1.73 m^2^ received SIR at target trough levels of 4 to 8 ng/mL, achieved no later than four weeks after commencement, for six months. The primary endpoint was slope of eGFR and proteinuria within six months of treatment with SIR. Secondary endpoints included blood count, lipids, and other known side effects of SIR. Since there is no common clinically accepted dynamic cut-off level, we calculated patient-specific dynamics and 95% confidence intervals for regression slopes in a historic cohort. Safety was not assumed if the mean decline in renal function and the mean incline in proteinuria exceeded the 95% confidence interval. With this pilot study we proved our hypothesis that a single daily oral dose of SIR in these patients does not lead to a eGFR-decline lesser than −8.8 mL/min per 1.73 m^2^ within six months (one-sided), and it does not lead to an incline of the logarithm of the protein/creatinine ratio greater than 0.39 within six months (one-sided).

### Procedures for eliciting reports of and recording and reporting of adverse events and intercurrent illnesses

AE will be classified into serious or minor, expected or unexpected, study-related, possibly study-related, or not study-related, and reported to the competent authority by the investigator immediately after its discovery. Any SAE, defined as any untoward medical occurrence that at any dose results in death, is life-threatening, requires inpatient hospitalization or prolongation of existing hospitalization, results in persistent or significant disability or incapacity, is a congenital anomaly or birth defect, or requires intervention to prevent permanent impairment or damage, will be reported to the competent authority by the investigator immediately after its discovery.

## Discussion

Currently there is no curative treatment for ADPKD. All previously conducted clinical trials, with the exception of one recently published randomized controlled pilot study [21], could not demonstrate any clinically relevant effect of mTOR inhibition on cyst growth or kidney function (Interventions for retarding the progression of autosomal dominant polycystic kidney disease (ADPKD): A systematic review and meta-analysis. Bolignano D., 51st ERA-EDTA Congress, Amsterdam, unpublished data) With this study we will investigate the effects of pulsed mTOR inhibition on kidney function. To the best of our knowledge, this is the first trial to implement this innovative administration of SIR in patients with advanced ADPKD.

## Trial status

The first participant was enrolled on 28 April 2014 and recruitment is ongoing. The last patient is expected to be included in April 2017.

## Abbreviations

ADPKD: Autosomal polycystic kidney disease
AE: Adverse event
CeMSIIS: Center for Medical Statistics, Informatics, and Intelligent Systems
CKD-MBD: Chronic kidney disease - mineral and bone disorder
CYP: Cytochrome p-450
eCRF: Electronic case report form
eGFR: Estimated glomerular filtration rate
EVER: Everolimus
KDIGO: Kidney disease improving global outcomes
MDRD: Modification of diet in renal disease
mTOR: Mammalian target of rapamycin
mTOR-I: Mammalian target of rapamycin inhibitor
P-gp: P-glycoprotein
PKD: Polycystic kidney disease
PTH: Parathyroid hormone
SAE: Severe adverse event
SIR: Sirolimus
